# Binocular dynamic visual acuity in dry eye disease patients

**DOI:** 10.3389/fnins.2023.1108549

**Published:** 2023-03-08

**Authors:** Xiaotong Ren, Yuexin Wang, Tingyi Wu, Dalan Jing, Xuemin Li

**Affiliations:** Department of Ophthalmology, Peking University Third Hospital, Beijing, China

**Keywords:** dynamic visual acuity (DVA), dry eye disease (DED), meibomian gland area, meibomian gland dropout, artificial intelligence (AI)

## Abstract

**Purpose:**

To investigate binocular dynamic visual acuity (DVA) for patients with dry eye disease (DED).

**Methods:**

The prospective study included DED patients. The binocular DVA at 40 and 80 degrees per second (dps), Ocular Surface Disease Index (OSDI), tear meniscus height (TMH), tear film break-up time first (TBUTF), corneal fluorescein staining (CFS), eyelid margin abnormalities and meibomian gland (MG) abnormalities morphology and function were evaluated. A deep learning model was applied to quantify the MG area proportion. The correlation between DVA and DED parameters was analyzed.

**Results:**

A total of 73 DED patients were enrolled. The age, OSDI, CFS, MG expressibility, secretion quality, and eyelid margin abnormalities were significantly positively correlated with the DVA for 40 and 80 dps (all *P* < 0.05). The MG area proportion in the upper eyelid was negatively correlated with DVA at 40 dps (*R* = −0.293, *P* < 0.001) and at 80 dps (*R* = −0.304, *P* < 0.001). Subgroup analysis by MG grade demonstrated that the DVA of patients with severe MG dropout (<25% of the total area) was significantly worse than other mild and moderate groups, both in 40 and 80 dps (all *P* < 0.05). The patients with CFS showed worse 40 (*P* < 0.001) and 80 dps (*P* < 0.001) DVA than the patients without CFS.

**Conclusion:**

Binocular DVA is significantly associated with DED symptoms and signs. The DED patients with CFS and severe MG dropout and dysfunction have worse DVA.

## 1. Introduction

Dry eye disease (DED) is a multifactorial ocular surface disorder that affects millions worldwide (Craig et al., 2017; Stapleton et al., 2017). The Tear Film and Ocular Surface Society Dry Eye Workshop II (TFOS DEWS II) pointed out in 2017 that the central pathophysiological concept of DED is the loss of tear film homeostasis (Bron et al., 2017; Craig et al., 2017). The tear film plays an important role as the forefront refractive component of the eye (Craig et al., 2017; Wolffsohn et al., 2017). Decreased tear stability in DED patients causes ocular surface-related and vision-related symptoms, yet the routine static visual acuity might be normal (Benitez-Del-Castillo et al., 2017; Koh et al., 2018). The negative effect of these symptoms may even lead to depression, and affect work productivity, personal success and the economy (Tong et al., 2010; Uchino et al., 2014; Nichols et al., 2016; Stapleton et al., 2017).

Increasing research evaluated the impact of DED on vision-related daily activities. Proceedings of the Osmoprotection in Dry Eye Disease–Expert Opinion (OCEAN) group meeting proposed that in DED patients with normal conventional visual acuity, the difficulties with driving, reading and computer use might be related to impaired visual function (Benitez-Del-Castillo et al., 2017). Thus, the effect of DED on visual function has been paid increasing attention (Tong et al., 2010; Uchino et al., 2014; Mathews et al., 2017; Karakus et al., 2018a,b). Previous studies demonstrated that the DED is associated with deteriorated contrast sensitivity, higher order aberration (HOA), objective scattering index (OSI), and surface asymmetric index (SAI) (Goto et al., 2006; Kaido, 2018; Koh, 2018; Ma et al., 2020). The current visual function assessment mainly focuses on static vision, and certain limitations exist as static vision disturbance could not sufficiently demonstrate functional disability (Karakus et al., 2018a). Most objects we see in real-life have relative motion, so a favorable dynamic vision function is required for daily tasks (Kaido, 2018; Wu et al., 2021; Wang et al., 2022a,b). Dynamic visual acuity (DVA) describes the ability to identify the details of an object as it moves (Nakatsuka et al., 2006; Palidis et al., 2017; Wu et al., 2021). It could better reflect real-life situations vision and is more sensitive to visual disturbance and improvement (Kaido, 2018; Wang et al., 2022a). There are several methods for DVA testing (DVAT), commonly classified into static- and moving-optotypes DVATs (Wu et al., 2021). The latter test with screen demonstration has the advantage of accessibility, standardization and a short learning curve that is generally used in ophthalmology (Palidis et al., 2017; Wu et al., 2021). To the best of our knowledge, no study has investigated the impact of DED on DVA.

This study aims to evaluate the DVA in DED patients and investigate the influential factors that might affect DVA, including objective and subjective clinical dry eye parameters. The present research provides insight into the application of the DVA test to evaluate the functional vision of DED patients. With further improvement, the DVA test might facilitate the assessment and treatment of DED in patients with high demand for dynamic vision, including athletes and drivers.

## 2. Materials and methods

### 2.1. Participants

The present research is a prospective cross-sectional study and the protocol is approved by the Human Research and Ethics Committee of Peking University Third Hospital (approval number M2020431). The research was conducted adhered to the tenets of the Declaration of Helsinki. Informed consent was obtained from each patient before enrollment.

Consecutive patients who were diagnosed with DED were enrolled from October 2021 to December 2021. DED was diagnosed according to TFOS DEWS II in 2017 (Craig et al., 2017). The inclusion criteria included: (a) age 18 to 45 years; (b) a monocular best-corrected visual acuity (BCVA) of 1.0 (decimal) or more. Exclusion criteria consisted of (a) severe ocular surface diseases, lens abnormalities, glaucoma, uveitis, and retinal diseases; (b) history of intraocular surgery; (c) diseases that affect the free movement of the globe, such as obvious extraocular muscle abnormalities, including Thyroid associated ophthalmopathy (TAO) and so on; (d) conjunctivochalasis; (e) dry eye related systemic diseases, such as Sjogren’s syndrome, Stevens-Johnson syndrome, and rheumatism; (f) cognitive disorders; (g) other diseases or conditions unsuitable for this clinical trial judged by the researchers.

### 2.2. Evaluation index

All ophthalmologic examinations were performed under unchanged conditions by a single investigator (RXT) in the same examination room. To minimize the influence of the preceding test on the subsequent test, the clinical assessments of the enrolled patients were conducted in the following order: OSDI questionnaire, Ocular surface comprehensive analyzer, DVA and slit-lamp. An interval of 5 min was arranged between two different tests.

#### 2.2.1. Ocular surface disease index

Patients’ subjective symptoms were evaluated by the OSDI questionnaire, which included 12 questions and every item scored 0 to 4. OSDI = (sum of scores for all questions answered × 100)/(total number of answered questions × 4). It ranged from 0 to 100.

#### 2.2.2. Oculus Keratograph–Ocular surface comprehensive analyzer

To evaluate the meibomian gland (MG) dropout and the tear film, including the tear meniscus height (TMH) and tear film break-up time first (TBUTF), a non-invasive, Placido ring-based, ocular surface comprehensive analyzer (Keratograph 5 M; OCULUS, Wetzlar, Germany) was used. To avoid errors, the examination was repeated three times in each patient. The analyzer captured infrared photographs of the anterior segment of the eyes, enabling assessments of TBUTF, inferior TMH and the extent MG dropout. The superior and inferior margin of the tear meniscus was manually labeled and then the TMH was calculated automatically by the machine.

**FIGURE 1:**
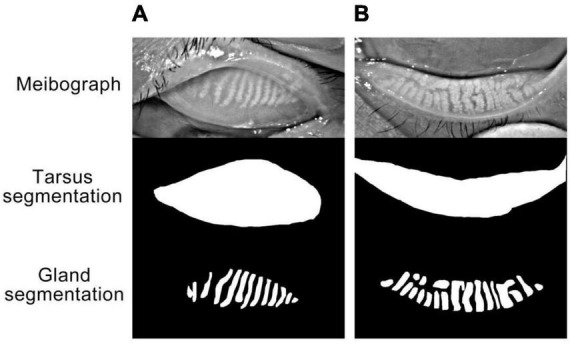
**(A,B)** Represented the upper eyelid and lower eyelid, respectively. “Meibograph” demonstrated the representatives of original infrared photographs obtained with OCULUS. The meibomian gland area (%) is calculated as the ratio of the meibomian gland and tarsus area. “Tarsus segmentation” and “Gland segmentation” represented the segmentation results of the tarsus and meibomian glands area from the meibograph by the deep learning model.

The meibograph with the best quality from three repeated capture was chosen for quantitative analysis using a deep learning model. The model leverages a convolutional network based on U-Net to segment the tarsus and meibomian glands area from meibograph. The model achieved an accuracy of 0.985 for segmenting the tarsus area and 0.937 for the meibomian gland area tested on an external dataset. The representatives of the meibograph and segmentation results for the upper and lower lid tarsus and meibomian gland area are demonstrated in Figure 1. The meibomian gland area proportion (%) is calculated as the ratio of the meibomian gland and tarsus area.

#### 2.2.3. Dynamic visual acuity test

We assessed binocular DVA (abbreviated simply as DVA) at 40 and 80 dps under the best-corrected visual acuity, and the steps have been described in detail in our previous research (Wang et al., 2022a,b). The DVA test system included a self-developed program which ran on a laptop and demonstrated a 14-inch 120 Hz TN screen. The testing program was programmed with MATLAB 2017b (MathWorks, Natick, MA, United States) to display the moving letter E (standard logarithm visual chart) of a certain speed and size. During the test, the letter E moved horizontally from the middle of the screen’s left side to its right side.

Before the test, we adjusted the seat to make the subject’s sight at the screen’s midpoint level and the test was performed at 4 m. Before the formal test, the subject was pre-trained sufficiently to understand the testing procedure and the motion pattern of the optotypes to avoid the learning effect. The test began with the optotypes three lines larger than the static visual acuity (SVA) result. Eight “letter E” optotypes with a random opening direction of the same size were presented once per 2 s. The subject was asked to identify the opening directions. If the accuracy reaches 5/8, we changed the optotypes to the smaller size until the size for which identified less than five optotypes. Recorded the minimum size (A, LogMAR) that subjects could recognize (five out of eight optotypes are identified correctly) and the number (b) of optotypes one size smaller that could be identified. The DVA calculation was as follows:


$$D\,V\,A=A-\frac{0.1}{8}*b$$

#### 2.2.4. Slit lamp examination

A slit lamp examination was performed to assess the eyelid margin signs, expressibility and secretion quality of the MG, corneal fluorescein staining (CFS). First, eyelid margin abnormalities were evaluated, including rounding of the posterior margin, irregularity of the eyelid margin, hyperkeratosis, eyelid margin telangiectasia and neovascularization. For each abnormality, if it existed, it would be recorded as 1 point; otherwise, it would be recorded as 0 points. Then the doctor applied fixed pressure to the glands at three positions (nasal, central, and temporal) of the upper and lower eyelid to evaluate the MG expressibility. In each position, five MGs were evaluated. MG expressibility was scored as 0, all 5 glands expressible; 1, 3–4 glands expressible; 2, 1–2 glands expressible; 3, no glands expressible. And it was calculated as the sum of the three positions with a total score of 9. Observe the secretion quality of eight MGs in the middle 1/3 of the upper and lower eyelids. The scoring criteria are as follows: 0, clear; 1, cloudy; 2, cloudy with debris; or 3, inspissated like toothpaste. Next, the doctor applied a drop of fluorescein sodium and then viewed the cornea with a slit lamp using cobalt blue illumination to assess CFS. The cornea was divided into 4 quadrants. Staining was scored from 0 to 3 in each quadrant and then summed. All the evaluation indexes were recorded with both eyes separately.

### 2.3. Statistical analysis

Statistical product and service solutions (SPSS) software version 23 (SPSS Inc., Chicago, IL, USA) was used for statistical analysis. The Kolmogorov-Smirnov test was applied to check the normality of the data distribution. The continuous variable data were presented as the mean and standard deviation (SD), and the categorical variables were shown as numbers (and percentages). The mean BCVA and each DED parameter are calculated from two eyes for subsequent correlation analysis. Pearson’s correlation was assessed to analyze the relationship between the DVA and age, BCVA, and each continuous evaluation index for DED. Otherwise, Spearman’s correlation analysis was applied. A multivariate linear model was applied to analyze the potentially influential factors for DVA, including the above factors in one model. Collinearity analysis was performed, and we excluded the variables with a variance inflation factor of more than five based on the clinical significance.

To further reveal the effect of MG dropout and CFS on DVA, patients were grouped based on the upper meibomian gland dropout severity in the original meibograph or the existence of CFS in subgroup analysis. Three groups were created, including mild MG dropout (>50% of the total area), moderate MG dropout (25–50% of the total area) and severe MG dropout (<25% of the total area), respectively. One-way ANOVA was used to compare the differences of DVA in 40 and 80 dps among groups with different meibomian gland dropout severity, and Bonferroni correction was conducted for *post-hoc* analysis. An Independent sample *t*-test was performed to compare the DVA between the DED patient with and without CFS. *P*-values less than 0.05 were considered statistically significant.

## 3. Results

### 3.1. The characteristics of the patients

Seventy-three patients were included in the study. The characteristics of the patients are summarized in Table 1. The average age of the enrolled patients was 35.57 ± 10.16 years. Male accounted for 26% of the enrolled patients.

**TABLE 1:** Characteristic of patients.

Parameters	Mean ± SD	Range
Age (years)	35.57 ± 10.16	20, 45
Sex (male/female)	19/54	
LogMAR BCVA	−0.039 ± 0.020	−0.1, 0
LogMAR DVA (40 dps)	0.109 ± 0.047	0.375, 0
LogMAR DVA (80 dps)	0.194 ± 0.079	0.425, 0.063
OSDI (scores)	43.28 ± 19.31	21, 81
TMH (cm)	0.17 ± 0.06	0.08, 0.30
TBUTF (s)	4.87 ± 2.43	2.0, 11
CFS (scores)	2.49 ± 2.38	0, 12
**Eyelid margin abnormalities**		
Rounding in posterior margin (number, %)	27 (37.0)	
Irregularity (number, %)	20 (27.4)	
Hyperkeratosis (number, %)	12 (16.4)	
Telangiectasia (number, %)	40 (54.8)	
**Meibomian gland assessments**		
Expressibility (upper, 0–9)	3.58 ± 1.32	2, 8
Expressibility (lower, 0–9)	2.25 ± 1.07	1, 6
Secretion quality (upper, 0–3)	1.63 ± 0.55	1, 3
Secretion quality (lower, 0–3)	1.24 ± 0.43	1, 3
MG area proportion (upper, %)	36.99 ± 14.04	0.45–66.43
MG area proportion (lower, %)	33.32 ± 12.36	2.18–65.98

BCVA, best-corrected visual acuity; CFS, corneal fluorescein staining; Dps, degree per second; DVA, dynamic visual acuity; MG, meibomian gland; OSDI, ocular surface disease index; TBUTF, tear film break-up time first; TMH, tear meniscus height.

### 3.2. The correlation between DVA and associated factors

The results of the correlation analysis between the DVA and the associated parameters are summarized in Table 2. The age, LogMAR BCVA, OSDI, and CFS were significantly positively correlated with the DVA for 40 dps and 80 dps (*P* < 0.05 for all the analyses). Except between hyperkeratosis of the eyelid margin and 40 dps DVA, there was a notable correlation between all the eyelid margin abnormalities and DVA, both 40 dps and 80 dps (*P* < 0.05 for all the analyses). A significant positive correlation was observed between the upper and lower meibomian gland expressibility, secretion quality and the DVA at 40 dps and 80 dps (*P* < 0.05 for all the analyses). The MG area proportion in the upper eyelid was significantly negatively correlated with the postoperative DVA at 40 dps (*R* = −0.293, *P* < 0.001) and at 80 dps (*R* = −0.304, *P* < 0.001).

**TABLE 2:** Correlation analysis between DVA and potential influencing factors.

Parameters	DVA at 40 dps	DVA at 80 dps
Age (years)	0.376**	0.294**
Sex	0.140	0.031
LogMAR BCVA	0.412**	0.259**
OSDI (scores)	0.382**	0.434**
TMH (cm)	0.020	0.003
TBUTF (s)	0.064	0.011
CFS (scores)	0.444**	0.428**
**Eyelid margin abnormalities**		
Rounding in posterior margin (0/1)	0.349**	0.500**
Irregularity (0/1)	0.393**	0.428**
Hyperkeratosis (0/1)	0.025	0.217**
Telangiectasia (0/1)	0.460**	0.457**
**Meibomian gland assessments**		
Expressibility (upper, 0–9)	0.361**	0.457**
Expressibility (lower, 0–9)	0.384**	0.473**
Secretion quality (upper, 0–3)	0.452**	0.553**
Secretion quality (lower, 0–3)	0.272**	0.297**
MGs area proportion (upper, %)	-0.293**	-0.304**
MGs area proportion (lower, %)	-0.161	-0.113

BCVA, best-corrected visual acuity; CFS, corneal fluorescein staining; Dps, degree per second; DVA, dynamic visual acuity; MG, meibomian gland; OSDI, ocular surface disease index; TBUTF, tear film break-up time first; TMH, tear meniscus height. P-values less than 0.05 were considered statistically significant. *P < 0.05, **P < 0.01.

### 3.3. Differences by MG grade and CFS

As shown in Table 3, there was no significant difference in age and BCVA among the three groups with different meibomian gland grades (all *P* > 0.05). The DVA at 40 dps of patients with severe meibomian gland dropout was significantly worse than that with mild (*P* < 0.001) and moderate (*P* = 0.001) meibomian gland dropout. There was no significant difference in 40 dps DVA between the mild and moderate meibomian gland group (*P* = 0.401). The result was similar for 80 dps DVA in that the severe meibomian gland dropout group had the worst DVA compared with the mild (*P* = 0.001) and moderate (*P* = 0.004) groups.

**TABLE 3:** Comparison of DVA among different Meibomian gland (MG) grades in the upper eyelid.

Parameters	Mild group	Moderate group	Severe group	P1	P2	P3
Number (patients)	25	26	22			
Sex (male/female)	6/19	8/18	5/17			
Age (years)	35.07 ± 7.60	36.51 ± 11.05	35.98 ± 10.89	0.300	0.069	0.291
LogMAR BCVA	0.024 ± 0.043	0.016 ± 0.039	0.013 ± 0.031	0.392	0.290	0.720
LogMAR DVA (40 dps)	0.088 ± 0.061	0.101 ± 0.057	0.155 ± 0.089	0.401	<0.001	0.001
LogMAR DVA (80 dps)	0.169 ± 0.063	0.187 ± 0.066	0.236 ± 0.095	0.292	0.001	0.004

Mild group: mild MG dropout (>50% of the total area), Moderate group: moderate MG dropout (25–50% of the total area), Severe group: severe MG dropout (<25% of the total area). DVA, dynamic visual acuity; dps, degree per second; BCVA, best-corrected visual acuity. P1: Mild group vs. Moderate group; P2: Mild group vs. Severe group; P3: Moderate group vs. Severe group. *P*-values less than 0.05 were considered statistically significant.

The result of DVA at 40 and 80 dps in DED patients with and without CFS is shown in Table 4. The CFS group showed significantly worse DVA, both at 40 (*P* < 0.001) and 80 dps (*P* < 0.001), than that in the non-CFS group.

**TABLE 4:** Comparison of DVA between CFS and non-CFS groups.

	Non-CFS group	CFS group	*P*
Number (patients)	40	33	
Sex (male/female)	11/29	8/25	
Age (Years)	36.361 ± 10.332	37.317 ± 9.722	0.084
LogMAR BCVA	0.012 ± 0.022	0.008 ± 0.024	0.168
LogMAR DVA (40 dps)	0.089 ± 0.072	0.138 ± 0.069	<0.001
LogMAR DVA (80 dps)	0.174 ± 0.079	0.223 ± 0.070	<0.001

BCVA, best-corrected visual acuity; CFS, corneal fluorescein staining; CFS group: CFS scores > 0 for any eye; dps: degree per second; DVA, dynamic visual acuity; Non-CFS group: CFS scores were 0 for both eyes. P-values less than 0.05 were considered statistically significant.

## 4. Discussion

This study aims to investigate the DVA in DED patients and investigate the associated influential factors. We found a significant correlation between DVA and DED severity in patients whose conventional static vision was normal. DVA serves as a linkage between DED parameters and dynamic visual ability, which could better reflect life scenes than static visual acuity. These observations indicate that it is necessary to test DVA in DED patients, especially for patients with high demand for dynamic vision. The present research innovatively proposes a simple and objective way to measure the dynamic vision in dry eye patients to better reflect daily life visual function.

Dry eye disease patients usually showed normal static visual acuity testing with a conventional visual chart. Therefore, although DED has been regarded as disturbing the quality of life, it has not been considered a serious visual disorder. Previous research found that the reading rate was lower in DED patients than in healthy control subjects (Ridder et al., 2013; Karakus et al., 2018b), and the reading rate decreased as the DED severity increased (Ridder et al., 2013; Ousler et al., 2015). The previous visual function evaluation on dry eye mainly focused on static vision assessment, including contrast sensitivity (CS), surface regularity index (SRI), surface asymmetry index (SAI), higher order aberrations (HOAs), objective scattering index (OSI), and potential visual acuity (PVA) (Huang et al., 2002; Goto et al., 2006; Puell et al., 2006; Ridder et al., 2009, 2013; Ousler et al., 2015; Benitez-Del-Castillo et al., 2017; Koh, 2018; Ma et al., 2020; Gao et al., 2021). These studies showed that tear film changes in DED patients might lead to irregularities on the corneal surfaces (Goto et al., 2006; Benitez-Del-Castillo et al., 2017; Koh et al., 2018; Ma et al., 2020) causing glare disability, irregular astigmatism, worse SRI, SAI, HOAs, PVA, and CS for the dry eye patients (Huang et al., 2002; Goto et al., 2006; Ridder et al., 2009). Irregular astigmatism, OSI and HOAs were correlated with the severity of DED (Goto et al., 2006; Benitez-Del-Castillo et al., 2017; Koh, 2018; Ma et al., 2020; Shimizu et al., 2020). And these optical quality indices in DED patients could improve after treatment (Huang et al., 2002; Qiu et al., 2012; Gao et al., 2021). However, considering the limitations of these static vision assessments, which could not sufficiently reflect life scenario visual function and these changes may be too subtle in mild dry eyes (Huang et al., 2002), the present study included dynamic vision assessment. Dynamic vision plays an important role in performing daily tasks (Ren et al., 2020) such as driving, speed reading, and identifying high-speed table tennis, and badminton. Deschamps et al. (2013) evaluated the impact of DED on visual performance while driving and pointed out that DED patients need more response time to identify target than healthy subjects and were more often missed targets appearing at a crossroad entrance. DVA represents visual acuity identifying the moving objects, and its special signaling pathway and influencing factors differ from static visual acuity (Rokszin et al., 2010; Skottun, 2016). Different eye movements were required for moving object detection. When we look at moving objects at a velocity of up to 50 degrees per second, smooth pursuits could stabilize it close to the fovea, but for higher velocity, saccades were needed to catch up with the gaze lag (de Brouwer et al., 2002; Palidis et al., 2017; Michel et al., 2020). Thus, we chose two velocities for the test, 40 and 80 degrees per second (dps) to better assess DVA for DED patients.

In the present study, we found that DVA was correlated with MG dropout and function, palpebral margin abnormalities, CFS and OSDI. There was a significant difference in DVA among patients with different degrees of meibomian gland dropout in the upper eyelid. The patients with severe MG dropout showed worse DVA than mild or moderate dropout in 40 and 80 dps. And DVA is correlated with the expressibility and secretion quality of MG. These results together indicated that meibomian gland morphology and function affect DVA. The obstruction of the meibomian gland and poor quality of secretion meibum contributes to the instability of the tear film and the rough optical surface (Mudgil and Millar, 2011), which might lead to a more prominent interface optical scatter (larger OSI). The obvious optical scatter of tear film might contribute to the greater artifact for moving object imaging, bringing worse DVA. In addition, the amount of meibum is significantly reduced due to severe dropout of the meibomian gland (Eom et al., 2013; Kim et al., 2018), and it also contributes to the instability of the tear film.

Similarly, corneal fluorescein staining (CFS) also posed a positive correlation with DVA. Past studies confirmed that CFS is related to visual quality (Kaido et al., 2011; Leonardi et al., 2019). Corneal staining shows corneal epithelial damage (Kaido et al., 2011), and the optical surface of the cornea becomes irregular, which might contribute to greater artifacts, and more inconsistent imaging when observing moving objects. In our research, eyelid margin abnormalities also showed relevance to DVA. The normal structural eyelid margin plays an important role in the distribution and formation of the normal tear film (Bai et al., 2021). The existence of eyelid margin abnormalities indicates more severe DED, the greater probability of tear film instability and higher CFS scores. Thus, the imaging of moving optotypes might be affected during the DVA test due to the rough optical surface.

Ocular Surface Disease Index sums up the subjective symptoms of DED and could reflect the severity of dry eye (Belmonte et al., 2017). The present study proved that worse DVA is associated with worse OSDI. The worse OSDI score, indicating uncomfortable DED-related visual experience, might cause shorter fixation time and more frequent blinking. An excellent DVA is closely related to reasonable eye tracking on moving objects. Problems such as non-persistence vision would affect the tracking of moving objects, including smooth pursuit and saccade, and reduce the prediction of moving object trajectory, leading to poor DVA.

The low TMH, indicating the quantitative deficiency of tear film, was associated with functional visual acuity and optical quality reduction (Goto et al., 2003; Kaido et al., 2006, 2011; Kaido, 2018). But no correlation was found between DVA and TMH in the present study. The quantity deficiency might not affect the stability and consistency of the tear film, which affects DVA. Short TBUTF contributes to impaired visual function (Kaido, 2018; Koh et al., 2018) but no correlation was found between DVA and TBUTF in our study. The individual data demonstrates that the shortest TBUT of the enrolled subjects was 2 s. It exceeded the time of the visual target moving from left to right. Therefore, the tear film might not break during the visual target movement.

Dynamic visual acuity could be recommended for evaluation for DED patients beyond conventional visual examinations and the evaluation of optical quality. The present research demonstrated that DVA is an excellent bridge linking visual function and dry eye symptoms and signs. Additionally, DVA is a sensitive indicator to detect the severity changes of dry eye. Severe MG dropout patients might be incompetent in some daily tasks for their poor DVA, including sports and driving. It is also helpful to determine the therapeutic effects of DED treatments, assessing the ability to return to normal visual function and performance of daily tasks. For the tasks with high demand for DVA, more attention should be paid to the DED. The DED in athletes might affect their sports level and the DED of drivers or pilot might affect their driving safety. The dynamic vision-based test could facilitate the comprehensive DED evaluation in these occupations. With further improvement and popularization, occupational-related DVA thresholds would establish and guide the DED treatment.

Certain limitations exist in the present study. The sample size is relatively small, and the research lacks a control group without DED for comparison. In addition, we did not classify DED types. Then, we only included subjects aged 18–45 in this study, whose disease severity might be mild. In the future, we will expand the age range of the subjects. Furthermore, the effect of dry eye treatment on DVA requires further exploration. Finally, the DVA test only involved one test distance and a single horizontal moving pattern. Improvements are planned to include different motor patterns for assessment. Future studies will include a larger sample, parameters for real-life tasks in more detail, and assessment before and after DED treatment.

## 5. Conclusion

In conclusion, binocular DVA is significantly associated with DED symptoms and signs. The DED patients with CFS and severe MG dropout and dysfunction have worse DVA. The present research provides the basis for the DVA test in DED evaluation. With further improvement, the DVA test might guide the DED assessment and treatment, especially in patients with high demand for dynamic vision.

## Data availability statement

The datasets generated and analyzed during the current study are not publicly available but are available from the corresponding author on reasonable request.

## Ethics statement

The studies involving human participants were reviewed and approved by the Human Research and Ethics Committee of Peking University Third Hospital. The patients/participants provided their written informed consent to participate in this study.

## Author contributions

XR: research design, data acquisition, and manuscript preparation. YW: research design, data analysis, and manuscript preparation. TW: data acquisition and data analysis. DJ: data acquisition and manuscript preparation. XL: research design. All authors read and approved the final version of this manuscript.
